# Development and validation of a nomogram to evaluate the therapeutic effects of second-line axitinib in patients with metastatic renal cell carcinoma

**DOI:** 10.3389/fonc.2023.1071816

**Published:** 2023-02-15

**Authors:** Dengqiang Lin, Peng Lai, Wen Zhang, Jinglai Lin, Hang Wang, Xiaoyi Hu, Jianming Guo

**Affiliations:** ^1^ Department of Urology, Zhongshan Hospital (Xiamen Branch), Fudan University, Xiamen, China; ^2^ Department of Traditional Chinese Medicine, Zhongshan Hospital, Fudan University, Shanghai, China; ^3^ Department of Urology, Zhongshan Hospital, Fudan University, Shanghai, China

**Keywords:** renal cell carcinoma, tyrosine kinase inhibitor, axitinib, nomogram, receiver operating characteristic

## Abstract

The unpredictable biological behavior and tumor heterogeneity of metastatic renal cell carcinoma (mRCC) cause significant differences in axitinib efficacy. The aim of this study is to establish a predictive model based on clinicopathological features to screen patients with mRCC who can benefit from axitinib treatment. A total of 44 patients with mRCC were enrolled and divided into the training set and validation set. In the training set, variables related with the therapeutic efficacy of second-line treatment with axitinib were screened through univariate Cox proportional hazards regression and least absolute shrinkage and selection operator analyses. A predictive model was subsequently established to assess the therapeutic efficacy of second-line treatment with axitinib. The predictive performance of the model was evaluated by analyzing the concordance index and time-dependent receiver operating characteristic, calibration, and decision curves. The accuracy of the model was similarly verified in the validation set. The International Metastatic RCC Database Consortium (IMDC) grade, albumin, calcium, and adverse reaction grade were identified as the best predictors of the efficacy of second-line axitinib treatment. Adverse reaction grade was an independent prognostic index that correlated with the therapeutic effects of second-line treatment with axitinib. Concordance index value of the model was 0.84. Area under curve values for the prediction of 3-, 6-, and 12-month progression-free survival after axitinib treatment were 0.975, 0.909, and 0.911, respectively. The calibration curve showed a good fit between the predicted and actual probabilities of progression-free survival at 3, 6, and 12 months. The results were verified in the validation set. Decision curve analysis revealed that the nomogram based on a combination of four clinical parameters (IMDC grade, albumin, calcium, and adverse reaction grade) had more net benefit than adverse reaction grade alone. Our predictive model can be useful for clinicians to identify patients with mRCC who can benefit from second-line treatment with axitinib.

## 1 Introduction

Globally, approximately 85% of renal tumors were renal cell carcinoma (RCC), which is one of the ten most common cancer types and characterized by unpredictable biological behavior and heterogeneity (1, 2). Until recently, surgical resection was the standard of care, with a favorable overall prognosis for patients with localized RCC. The 5-year survival rate for patients with early stage I and II/III RCC are 93% and 72.5%, respectively, whereas those for patients with stage IV metastatic RCC is 12% (3). Moreover, 17%–30% of patients present with advanced stage of the disease at primary diagnosis, and 20%–40% of patients with localized disease eventually develop advanced disease (4, 5), which requires systemic therapies. In the past decades, the therapeutic strategy for locally advanced and metastatic RCC (mRCC) has broadened remarkably—from the use of cytokines (interferon-alpha and interleukin-2) to the administration of molecular-targeted therapies, such as tyrosine kinase inhibitors (TKIs) (6). Although treatment with molecular-targeted therapies has improved the prognosis of patients with mRCC, first-line therapies fail in most patients because of disease progression or unacceptable side effects (7).

After first-line therapies fail, a second-line therapeutic strategy is selected to improve patient prognosis. According to the NCCN guidelines, axitinib is recommended as a second-line treatment option. Compared with sorafenib as second-line treatment, axitinib significantly increased median progression-free survival (PFS) time and provided a better objective response rate for patients with mRCC who received sunitinib or cytokine treatment as a first-line therapy in a randomized phase III study (AXIS trial) (8). Moreover, the results of subgroup analyses of the AXIS study attested to the efficacy of axitinib in the Asian population, further supporting the registration of axitinib in China (8). Axitinib is more cost-effective than sorafenib (9). By contrast, a retrospective and noncomparative phase II trial indicated that the 5-year survival rate of patients who received axitinib was 20.6% after failure of prior systemic treatment (10). The differences in PFS and overall survival were insignificant in patients with mRCC who received axitinib or everolimus as second-line treatment (11); however, axitinib had a manageable tolerability profile.

Genomic studies have reported intratumoral and intertumoral heterogeneity in RCC (8, 12, 13), which leads to differential prognosis and response to targeted treatment. Consequently, it is imperative to screen patients with mRCC who can benefit from axitinib therapy after failure of first-line therapies and improve the cost-effectiveness of therapy. This study aimed to retrospectively evaluate the prognostic clinicopathological parameters associated with the therapeutic effects of second-line treatment with axitinib.

## 2 Methods

### 2.1 Patients and inclusion criteria

**Figure 1:**
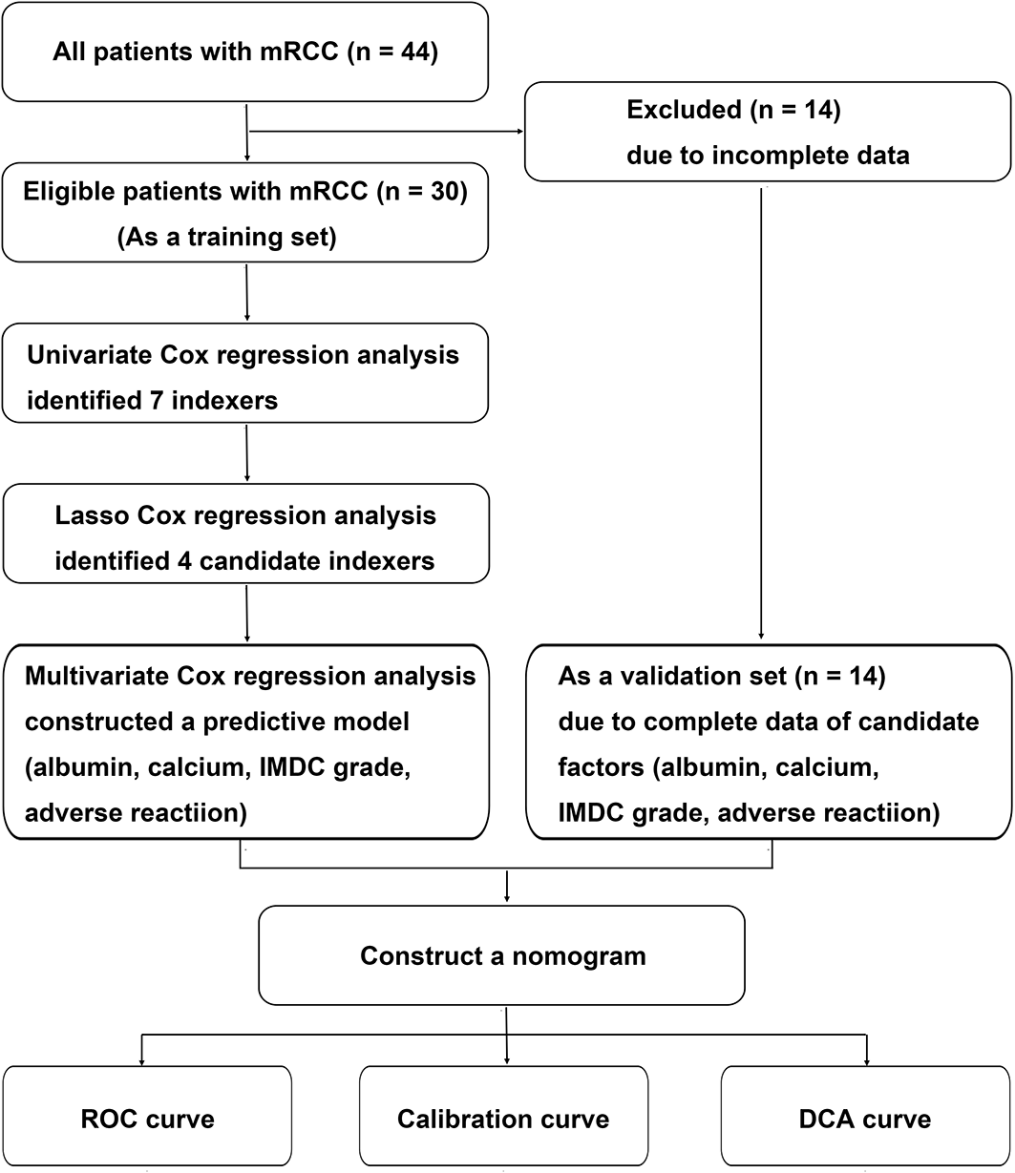
Flow chart of the data selection and process. IMDC, International Metastatic Renal Cell Carcinoma Database Consortium classification; LASSO, least absolute shrinkage and selection operator; RCC, renal cell carcinoma.

The study was conducted in accordance with the Declaration of Helsinki (revised in 2013). Study approval was given by the Ethics Committee of Zhongshan Hospital, affiliated to Fudan University, China (B2016-030). Data from 44 patients with advanced RCC, who received axitinib as second-line targeted therapy between December 2014 and December 2021 at the Department of Urology, Zhongshan Hospital, Fudan University, were retrospectively collected and analyzed. The inclusion criteria were as follows: (1) advanced RCC or mRCC verified histopathologically with surgery or biopsy, (2) advanced RCC irrespective of pathological type, (3) advanced RCC irrespective of first-line treatment, and (4) advanced RCC with axitinib as second-line targeted therapy. Because 14 patients lacked complete clinicopathological data, 30 patients were finally enrolled in the study as the training set to evaluate factors related to the therapeutic effects of second-line treatment with axitinib and construct a predictive model. Four clinical parameters, namely albumin, calcium, International Metastatic RCC Database Consortium (IMDC) grade, and adverse reaction grade, were further identified. Because complete data were available for the four candidate factors for 14 patients, they were included in the validation set to verify the model (**Figure 1**). Biochemical parameters were collected before patients received axitinib.

### 2.2 Statistical analysis

Statistical analysis was performed using SPSS v23 and R v4.20. Continuous variables are presented as the mean and standard deviation, and categorical variables are presented as frequency or percentage. Comparisons of continuous variables between two groups were performed with the *t* test, and categorical variables were compared using the chi-square test or Fisher’s exact test. p-Value < 0.05, two-tailed, was considered statistically significant. Kaplan–Meier survival plots were generated with the log-rank statistic using the survival package of R.

We first screened the clinicopathological parameters associated with the therapeutic effects of second-line treatment with axitinib using univariate Cox proportional-hazards regression (CPHR) analysis. Because CPHR is not used to analyze multidimensional survival datasets, the least absolute shrinkage and selection operator (LASSO) technique was subsequently performed for variable selection and shrinkage from many clinical variables identified by univariate CPHR, using the glmnet package of R (8, 14). Finally, we identified and then established a predictive model based on four clinical parameters (albumin, calcium, IMDC grade, and adverse reaction grade) through multivariate CPHR.

To evaluate the predictive accuracy of the model, time-dependent receiver operating characteristic curve (ROC) and area under curve (AUC) at 3-, 6-, and 12-month PFS after axitinib treatment were constructed using the survival package of R. Concordance index (C-index) is used to evaluate predictive accuracy (15). The consistence between predicted PFS probability and actual PFS probability was confirmed using a calibration curve after 1000 bootstrap resampling. The ROC curve and AUC are not used to make clinical decisions. In clinical practice, decision curve analysis (DCA) was used to estimate the net benefit for patients based on threshold from the predictive model.

## 3 Results

### 3.1 General characteristics

Based on the inclusion criteria, 44 patients with advanced RCC received axitinib as a second-line targeted therapy. Thirty patients were included in the training set and 14 in the validation set. The clinicopathological features of patients are shown in **Table 1**. Mean age at initial diagnosis was 60.25 ± 10.02 years. Most of the patients were men (79.55%, 35/44) and had received nephrectomy (86.36%, 38/44). The pathologic type of 35 patients (79.55%) was clear cell carcinoma, of which two tissue samples were associated with sarcomatous degeneration. The maximum tumor diameter was 2.5–15.0 cm. The most common metastatic sites were the lungs (63.64%, 28/44), bones (34.10%, 15/44), and liver (15.91%, 7/44). In addition, the lymph node was a common distant site (40.91%, 18/44). In some patients, the tumor metastasized to the brain (4.55%, 2/44), adrenal gland (4.55%, 2/44), and pancreas (9.10%, 4/44). Mean overall follow-up time was 1485.44 ± 1150.61 days and median survival time was 2071 days for the whole cohort.

**Table 1:** Patient demographics and clinicopathological features.

Variable	All patients (n = 44)	Training set (n = 30)	Validation set (n = 14)	p-Value
**Age (years)**	60.25 ± 10.02	59.37 ± 10.91	62.14 ± 7.77	0.3990
**Gender**				0.2471
Male (n)	34	25	9	
Female (n)	10	5	5	
**Tumor location**				0.5206
Left	21	13	8	
Right	23	17	6	
**Nephrectomy**	38/44	25/30	13/14	0.6467
**Pathologic type**				0.6951
CCRCC	36	25	11	
nCCRCC	8	5	3	
**Sarcomatous change**	2/44	1/30	1/14	0.5720
**Primary tumor size (cm)**	6.91 ± 2.94	6.87 ± 3.13	7.00 ± 2.59	0.8932
Metastasis				
Liver	7/44	3/30	4/14	0.1167
Lung	28/44	20/30	8/14	0.5408
Bone	15/44	9/30	6/14	0.4020
Brain	2/44	2/30	0/14	0.3227
Lymph node	18/44	12/30	6/14	0.8575
Other	25/44	14/30	11/14	0.0466
**First-line drug**				0.5009
Sorafenib	5	3	2	
Sunitinib	33	24	9	
Pazopanib	6	3	3	
**Time from first- to second-line treatment (days)**	841.68 ± 695.15	647.43 ± 593.93	662.57 ± 712.80	0.9419
**Results of first-line treatment**				0.7530
PD	25	18	7	
SD	17	11	6	
PR	2	1	1	
**Time from second-line treatment (days)**	446.02 ± 350.21	486.30 ± 372.28	359.71 ± 291.11	0.2690
**Results of second-line treatment**				0.5881
PD	21	15	6	
SD	17	12	5	
PR	6	3	3	
**KPS score >80**	31	21	10	>0.9999
**Hemoglobin**	116.07 ± 23.94	120.77 ± 20.67	104.33 ± 28.28	0.0429
**Platelet**	237.31 ± 117.35	241.30 ± 119.25	226.45 ± 116.90	0.7244
**Lymphocyte**	1.53 ± 0.80	1.59 ± 1.06	1.53 ± 1.12	0.8712
**Neutrophil**	3.62 ± 2.26	3.22 ± 1.53	4.70 ± 3.46	0.0629
**C-reactive protein**	9.59 ± 22.87	4.69 ± 10.72	38.04 ± 33.40	0.0002
**Creatinine**	127.21 ± 34.43	132.43 ± 35.31	114.17 ± 29.52	0.1218
**Albumin**	28.68 ± 6.07	38.60 ± 5.57	40.17 ± 5.87	0.3967
**Calcium**	2.45 ± 0.25	2.47 ± 0.16	2.44 ± 0.43	0.7366
**IMDC grade**				0.6824
I	6	5	1	
II	30	20	10	
III	8	6	2	
**Largest adverse reaction grade**				0.8266
0	5	3	2	
I	16	12	4	
II	22	15	7	
III	1	1	0	
**Follow-up time (days)**	1485.44 ± 1150.61	1623.83 ± 1268.75	1166.08 ± 764.94	0.2353
**Alive at last follow-up**	22/44	16/30	6/14	0.5174

CCRCC, Clear cell renal cell carcinoma; nCCRCC, non-clear cell renal cell carcinoma; PD, Progressive disease; PR, Partial response; SD, Stable disease; KPS score, Karnofsky score; IMDC, International Metastatic Renal Cell Carcinoma Database Consortium classification.

Axitinib was introduced as a second-line targeted therapy after the failure of first-line treatment with drugs, including sorafenib (n = 5), sunitinib (n = 33), and pazopanib (n = 6). Failure of first-line therapy was a result of progression (25/44, 56.82%) or intolerable adverse effects (19/44, 43.18%). Mean therapeutic time and median PFS time of first-line treatment were 841.68 ± 695.15 days and 1058 days, respectively, for the whole cohort. Time of disease progression during second-line treatment with axitinib was defined as the time from the start of axitinib treatment to the first documentation of progression. Patients during treatment comprised the progression (Pro) group (n = 20) or the progression-free (ProFree) group (n = 24). The mean therapeutic time of second-line treatment was 446.02 ± 350.21 days for the whole cohort and 486.30 ± 372.28 days and 359.71 ± 291.11 days for the training and validation sets, respectively (p = 0.2690).

Statistically significant differences were present between the training and validation sets within the cohort, including hemoglobin level (120.77 ± 20.67 vs. 104.33 ± 28.28 g/L, p = 0.0429), C-reactive protein level (4.69 ± 10.72 vs. 38.04 ± 33.40 mg/L, p = 0.0002), and other metastatic sites (14/30 vs. 11/14, *P* = 0.0466). The differences were not statistically significant for the other clinicopathological features. However, hemoglobin level, C-reactive protein level, and other metastatic sites were unrelated to the therapeutic effects of second-line treatment with axitinib.

### 3.2 Subtype analysis

Results of subtype analysis are shown in **Table 2**. Albumin concentration was higher in the Pro group than in the ProFree group (41.13 ± 4.64 vs. 35.75 ± 6.39 g/L, p = 0.0024), and patients with mRCC who were malnourished (albumin ≤35 g/L) were more likely to have disease progression (8/10 vs. 12/34, p = 0.0270). Patients who were younger (<75 years old) did not benefit more from second-line treatment with axitinib than patients who were older (≥75 years old, p = 0.5832). Age distribution between the Pro and ProFree groups was not different (58.10 ± 11.14 vs. 62.04 ± 8.81 years, p = 0.1972). Higher levels of calcium (≥ 2.55 mmol/L) were related to worse prognosis than lower levels (<2.55 mmol/L) (9/12 vs. 11/32, p = 0.0212). Nephrectomy in patients with RCC did not affect the therapeutic effect of second-line treatment with axitinib. Consistently, significant differences between first-line treatment settings or metastatic sites and efficacy of second-line treatment with axitinib were not verified.

**Table 2:** Subtype analysis.

Variable	Difference of axitinib efficacy		p-Value
	Progression	Progression-Free	
**Age (years)**	58.10 ± 11.14	62.04 ± 8.81	0.1972
**Age ≥75 years**			0.5832
Yes	2	1	
No	18	23	
**Gender**			0.7344
Male	16	18	
Female	4	6	
**Tumor location**			>0.9999
Left	10	11	
Right	10	13	
**Nephrectomy**			>0.9999
Yes	17	21	
No	3	3	
**Pathologic type**			0.4361
CCRCC	15	21	
nCCRCC	5	3	
**Sarcomatous change**			0.4926
Yes	0	2	
No	20	22	
**Primary tumor size ≤7 cm**			0.5385
Yes	11	16	
No	9	8	
**Liver metastasis**			0.2172
Yes	5	2	
No	15	22	
**Lung metastasis**			>0.9999
Yes	13	15	
No	7	9	
**Bone metastasis**			0.2097
Yes	9	6	
No	11	18	
**Brain metastasis**			>0.9999
Yes	1	1	
No	19	23	
**Lymph node metastasis**			0.2268
Yes	6	12	
No	14	12	
**Other metastasis sites**			0.1151
Yes	9	16	
No	11	6	
First-line drug			
**Sorafenib**			0.1605
Yes	4	1	
No	16	23	
**Sunitinib**			0.4728
Yes	14	19	
No	6	4	
**Pazopanib**			0.6731
Yes	2	4	
No	18	20	
**KPS score >80**			0.5220
Yes	13	18	
No	7	6	
**Albumin (g/L)**	35.75 ± 6.39	41.13 ± 4.64	0.0024
**Albumin ≤35 g/L**			0.0270
Yes	8	2	
No	12	22	
**Calcium**	2.49 ± 0.22	2.41 ± 0.28	0.3053
**Calcium ≥2.55 mmol/L**			0.0212
Yes	9	3	
No	11	21	
**IMDC grade I**			0.1977
Yes	1	5	
No	19	19	
**Largest adverse reaction grade ≤I**			>0.9999
Yes	10	11	
No	10	13	

CCRCC, Clear cell renal cell carcinoma; nCCRCC, Non-clear cell renal cell carcinoma; PD, Progressive disease; PR, Partial response; SD, Stable disease; KPS score, Karnofsky score; IMDC, International Metastatic Renal Cell Carcinoma Database Consortium classification.

### 3.3 Prognostic model construction

**Figure 2:**
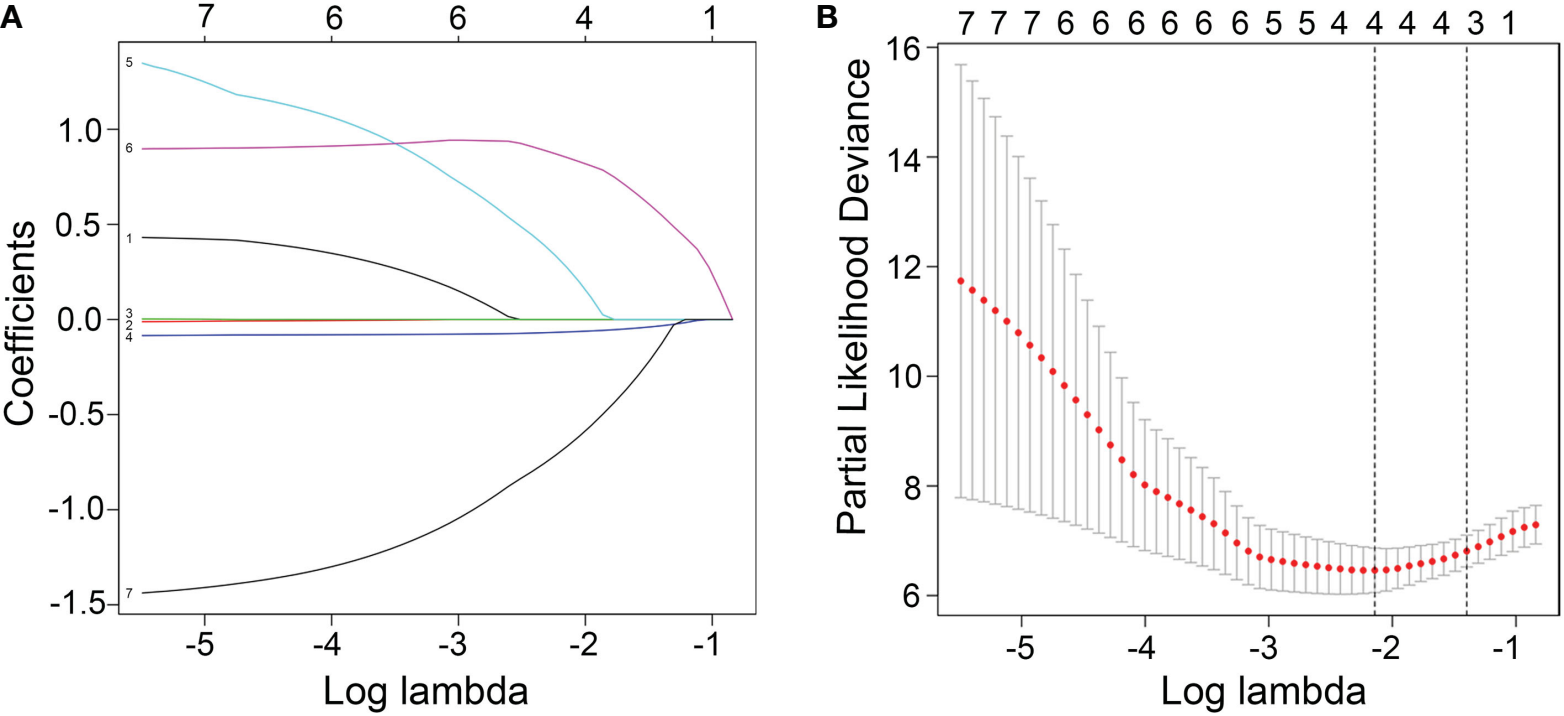
**(A)** Screening path of the least absolute shrinkage and selection operator (LASSO) regression model. **(B)** Penalty parameter (log lambda) in the LASSO regression model.

**Figure 3:**
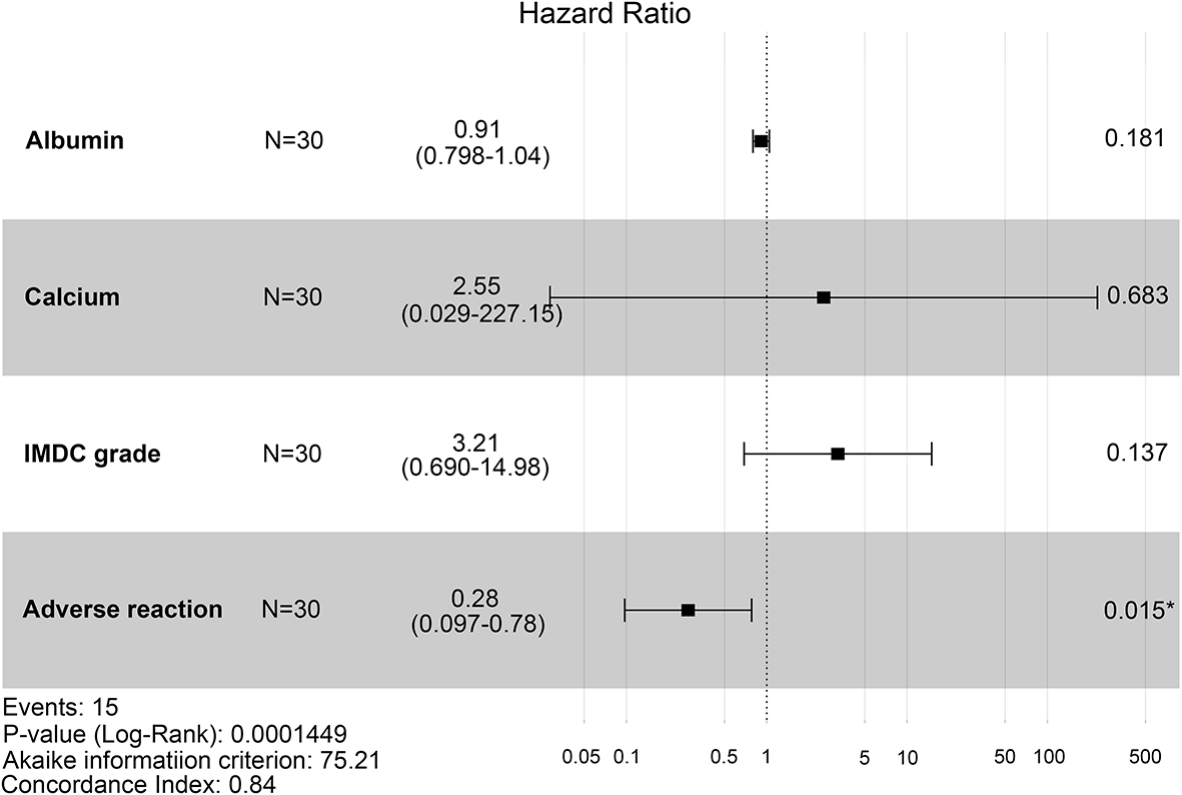
Forest plot of hazard ratios for four clinicopathological features (IMDC grade, albumin, calcium, and adverse reaction) using multivariate Cox proportional-hazards regression analysis. IMDC: International Metastatic Renal Cell Carcinoma Database Consortium classification.

**Figure 4:**
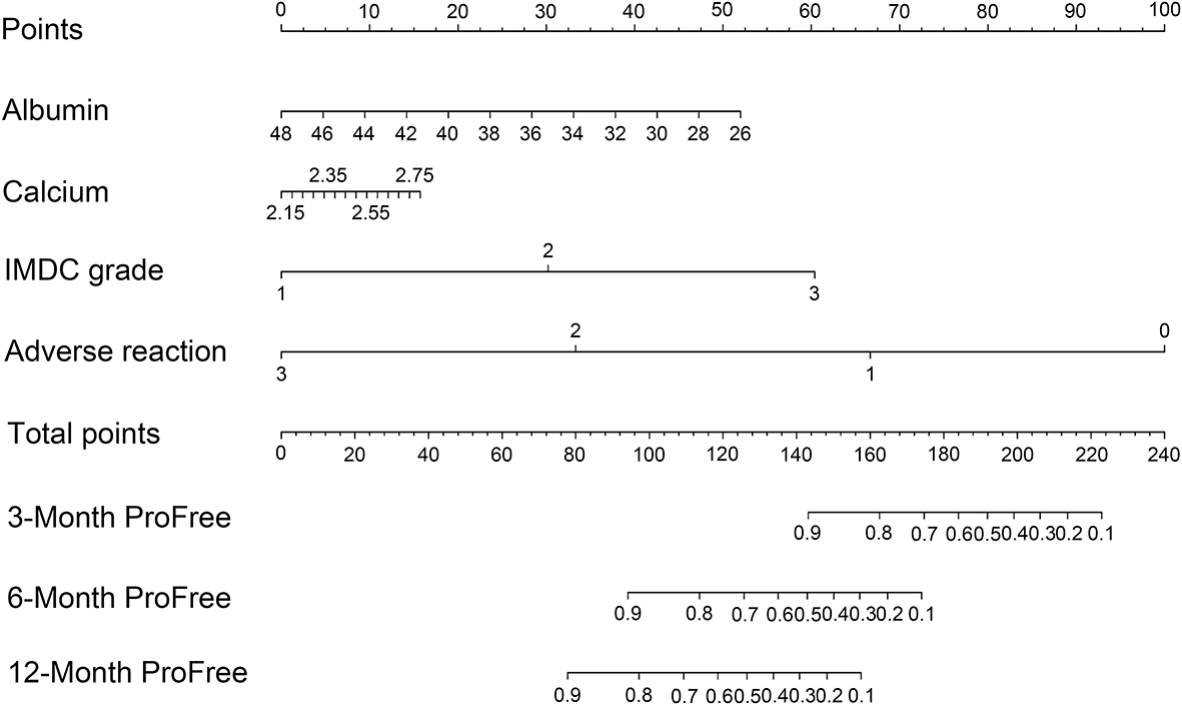
Nomogram based on the logarithm of four clinicopathological features (IMDC grade, albumin, calcium, and adverse reaction) predicting the efficacy of second-line treatment with axitinib in patients with metastatic renal cell carcinoma after failure of prior systemic treatment. IMDC: International Metastatic Renal Cell Carcinoma Database Consortium classification, ProFree: progression-free.

To evaluate the therapeutic effects of second-line treatment with axitinib, univariate CPHR analysis was used to identify potentially important factors. Seven parameters were screened, namely, IMDC grade [hazard rate (HR) = 5.26, p < 0.0001], albumin (HR = 0.82, p < 0.0001), calcium (HR = 172.34, p = 0.0005), adverse reaction grade (HR = 0.31, p = 0.0169), Karnofsky score (KPS score, HR = 0.92, p = 0.0442), bone metastasis (HR = 2.85, p = 0.0462), and hemoglobin (HR = 0.97, p = 0.0124) (**Table 3**). These parameters were incorporated into LASSO regression analysis to avoid bias from collinearity between factors (**Figure 2**). IMDC grade, albumin, calcium, and adverse reaction grade, with non-zero coefficients, were further enrolled in multivariate CPHR analysis to construct a prognostic model. IMDC grade had the highest hazard ratio (HR) (3.21, p = 0.1370), followed by calcium (2.55, p = 0.6833) (**Figure 3** and **Table 2**). Adverse reaction grade was an independent prognostic index that correlated with the therapeutic effects of second-line treatment with axitinib. To construct a quantitative and more intuitive tool for the individualized prediction of the therapeutic effects of second-line treatment with axitinib in patients with advanced RCC, a novel prognostic nomogram was established based on the four parameters, and the probability of 3-, 6-, and 12-month PFS was predicted (**Figure 4**).

**Table 3:** Results of the Cox proportional-hazards regression analysis.

Variable	Univariate CPHR		Multivariate CPHR	
	HR	p-Value	HR	p-Value
**IMDC grade**	5.26	<0.0001	3.21	0.1370
**Albumin**	0.82	<0.0001	0.91	0.1814
**Calcium**	172.34	0.0005	2.55	0.6833
**Adverse reaction**	0.31	0.0169	0.28	0.0152
**KPS score**	0.92	0.044		
**Bone metastasis**	2.85	0.046		
**Hemoglobin**	0.97	0.0124		

CPHR, Cox proportional-hazards regression; HR, hazard ratio; KPS score: Karnofsky score; IMDC: International Metastatic Renal Cell Carcinoma Database Consortium classification.

### 3.4 Predictive performance of the model

**Figure 5:**
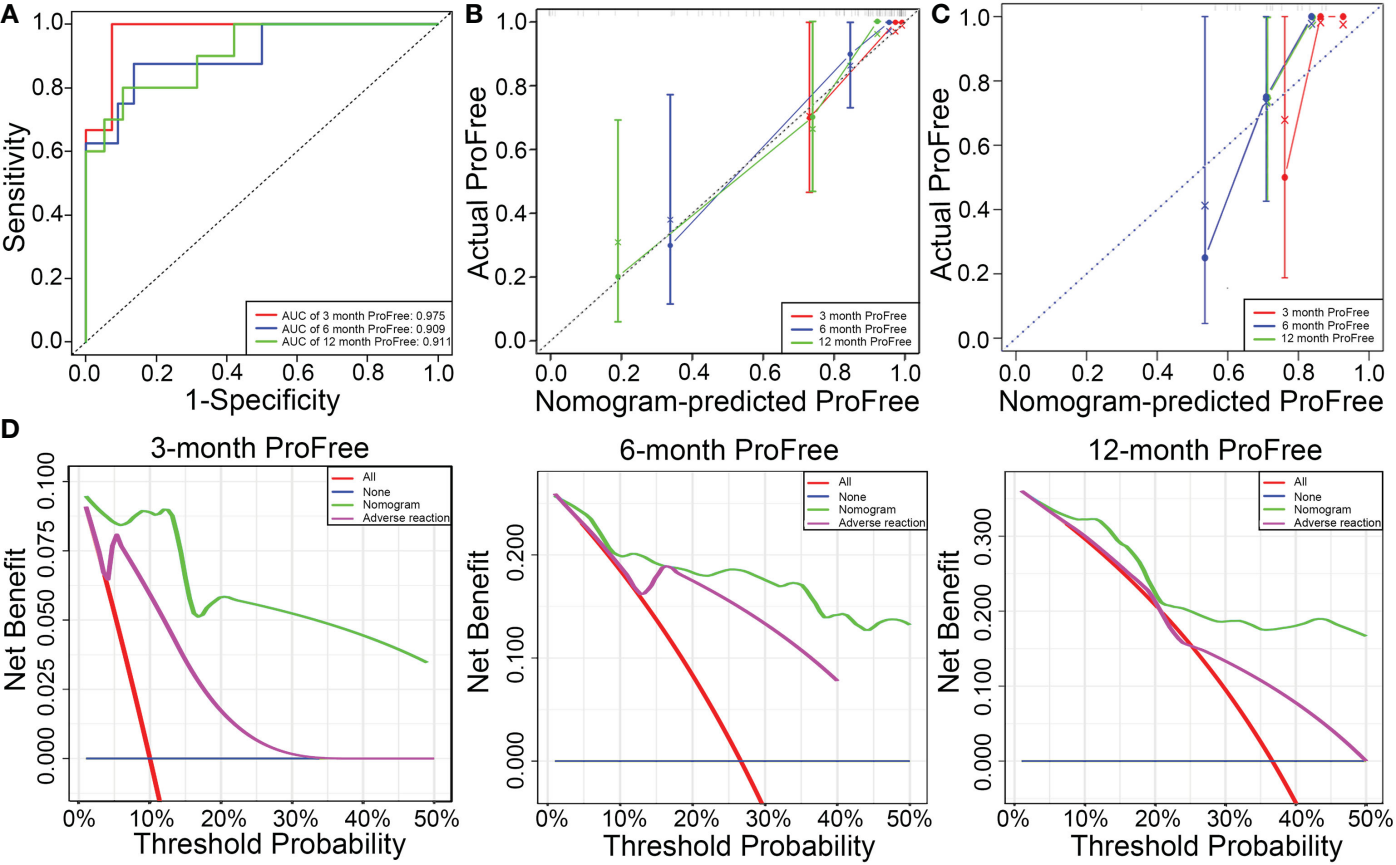
**(A)** Predictive performance of the model is evaluated using receiver operating characteristic curve. **(B)** Consistency of the model is evaluated using a calibration curve in the training set. **(C)** Consistence of the model is evaluated using a calibration curve in the validation set. **(D)** Decision curve analysis to evaluate the clinical benefit of 3-, 6-, and 12-month PFS and compare the clinical benefit of the model based on four parameters (IMDC grade, albumin, calcium, and adverse reaction) with adverse reaction grade.

The C-index value of the model was 0.84, suggesting that the predictive model had excellent predictive performance. Time-dependent ROC curve analysis verified the accuracy of the model; AUC values for the prediction of 3-, 6-, and 12-month PFS were 0.975, 0.909, and 0.911, respectively (**Figure 5A**). After 1000 bootstrap resampling was complete, the predictive model showed excellent consistency between predicted PFS probability and actual PFS probability at 3, 6, and 12 months, confirmed by the calibration curve (**Figure 5B**). The results were verified in the validation set, which had a C-index value of 0.776 (**Figure 5C**). Moreover, DCA was used to evaluate net benefit and make clinical decisions at 3, 6, and 12 months (**Figure 5D**). A nomogram (green) based on a combination of IMDC grade, albumin, calcium, and adverse reaction grade showed more area than adverse reaction grade alone (purple) (**Figure 5D**).

## 4 Discussion

Two primary signaling pathways are involved in RCC pathogenesis—vascular endothelial growth factor (VEGF) and mammalian target of rapamycin (mTOR) signaling pathways (16, 17). Loss mutation of the von Hippel–Lindau (VHL) is a common event in many RCCs, and then causes the abnormal activation of the above pathways, which is linked to cancer progression and poor prognosis (18). Currently, many TKIs targeting to VEGF-induced angiogenesis, including sunitinib, pazopanib, and axitinib, have been developed and are integral to the treatment (6, 16). However, RCC is characterized by a wide range of molecular and clinicopathological heterogeneity. Although considerable efforts have been made in the past decades to treat mRCC, targeted agents offer limited benefits to most patients. Compared with 8–9 months in the first-line treatment setting, the average time of stable disease is 5–6 months in the second-line treatment setting (19). At second-line treatment setting, axitinib significantly increased PFS time and improved objective response rate compared with sorafenib (8). Compared to first-line treatment with TKIs, axitinib not only showed fewer side effects, such as hepatotoxicity, hematological toxicity, and hypertension (20–22), but also immunomodulatory effects, where it downregulated the expression of the immune-suppressor signal transducer and activator of transcription 3 in patients with RCC (23), indicating that axitinib is relatively potent and must be further explored in combination therapy, first- or second-line setting.

However, fewer studies have identified biomarkers, including clinicopathological features and biochemical indices, to guide treatment. Biomarkers related to the therapeutic effects of second-line treatment with axitinib should be identified based on precision medicine or individual treatment.

In this study, a nomogram (C-index value = 0.84) was developed based on four variables (IMDC grade, albumin, calcium, and adverse reaction grade) in the test set. AUC values of the model for the prediction of 3-, 6-, and 12-month PFS were 0.975, 0.909, and 0.911, respectively. In addition, the model was internally validated after 1000-bootstrap resampling and externally validated in the validation set. But neither the ROC curve nor the calibration curve guides clinical decision. The DCA method was used to first evaluate the benefit of the predictive model and then help make a rational clinical decision. To our knowledge, DCA has never been used to evaluate the therapeutic effects of axitinib. Therefore, the performance of this prognostic model is reliable and accurate. Of course, small sample size is a limitation of this study. Moreover, independent validation sets from other centers were not enrolled in this study. Thus, further studies must verify the conclusion made using this prognostic model.

Hypertension is the most frequently documented adverse reaction in patients who received second-line treatment with axitinib (8); therefore, hypertension can be an effective predictor of axitinib efficacy. For instance, diastolic blood pressure ≥ 90 mmHg (23–26) and systolic blood pressure ≥ 140 mmHg (25, 27) were related to improved outcome of axitinib. Consistently, the findings of this study indicated that more adverse reaction grade was as an independent protective biomarker of axitinib efficacy. Compared with variable hypertension alone, the adverse reaction grade in this study reflected more information about the kinds of side effects, such as hypertension, fatigue, diarrhea, myelosuppression, hypothyroidism, and stomatitis. Moreover, blood pressure may be affected by many factors. In other words, its specificity is worse compared to our indexer that consists of all adverse reaction grades. However, it is still unclear for us and other researchers whether the adverse reaction, when it occurs, should be included into our nomogram, which must be further explored in prospective studies. Generally, 4 weeks is optimal for evaluating the efficacy and adverse reaction grade of second-line treatment with axitinib. Irrespective of the Memorial Sloan Kettering Cancer Center risk score or IMDC risk score, hypercalcemia in patients with mRCC was considered a risk factor for poor outcome, such as advanced stages and bone metastasis (28, 29). Consistently, in this study, IMDC grade and calcium level are included in the nomogram, confirming that higher IMDC grade and hypercalcemia are associated with lesser efficacy of axitinib. Albumin is sensitive to the nutritional state. Many studies have demonstrated that albumin is a risk parameter for the prognosis of some diseases, such as gastrointestinal stromal tumors, human immunodeficiency virus, lymphoma, and cutaneous malignant melanoma (30–35). For example, Datta et al. (31) reported that low albumin level was common in patients with stage IV cutaneous malignant melanoma. However, to our knowledge, the relationship between the prognosis of RCC or efficacy of TKIs and albumin concentration remains unclear. The findings of this study demonstrated that second-line axitinib treatment had worse efficacy in patients with RCC who were malnourished. Thus, improved nutrition may benefit more during targeted, second-line treatment with axitinib.

Older patients with RCC have often been excluded from receiving axitinib treatment, owing to safety concerns. According to Hideaki et al. (36), axitinib therapy was not only effective but also safe in patients aged >75 years. The results of this study revealed that patients aged <75 years old did not benefit more than patients aged ≥75 years (p = 0.5832). Patients in the ProFree group may be older than those in the Pro group (62.04 ± 8.81 vs. 58.10 ± 11.14 years, p = 0.1972), further suggesting that treatment with axitinib in older patients is worthy of attention. According to a phase III AXIS study, there was a significant difference for the effect size of the PFS benefit in different prior first-line treatments (37). In this study, differences in axitinib efficacy were not statistically significant between prior first-line treatment types.

This study has limitations. First, the sample size was small (n = 44), and the study was retrospective. Although the patients were divided into the training set and validation set, which was used to validate the performance of the model, the relatively small sample size and retrospective nature of the study significantly affected the accuracy and predictive performance of the study. Second, although patients were enrolled regardless of the type of first-line therapy, including sorafenib (n = 5), sunitinib (n = 33), and pazopanib (n = 6), patients who received a combination of TKI and immunotherapy as a first-line therapy were not included in the nomogram. Combined treatment with lenvatinib and pembrolizumab was related to significantly longer PFS and overall survival than that with sunitinib (38). Therefore, it is unclear whether the combination of TKI and immunotherapy as a first-line therapy could affect the efficacy of second-line treatment with axitinib. In addition, results from KEYNOTE 426 indicated that patients who received pembrolizumab–axitinib showed better ORR (59.3% vs. 35.7%) and median PFS (15.1 vs. 11.1 months) compared with patients who received sunitinib (39). Similarly, whether the model can be used to evaluate the efficacy of first-line treatment with axitinib, with a combination of pembrolizumab (39) or avelumab (20), is unclear.

Although the included parameters in the model may not only indirectly reflect plasma exposure of the drug by distinguishing adverse grade (23, 40) but also directly reflect individualized status, such as nutrition (albumin) and biochemical level (calcium), those parameters don’t reflect altered signaling pathways such as VHL, VEGF, mTOR, platelet-derived growth factor (PDGF), cell cycle, p53 Related Signaling, Ferroptosis, and so on (17, 41, 42). Additionally, imaging features of tumor during targeted therapy should be considered. The predictive performance and scope of applicability of the model to evaluate the efficacy of second-line axitinib treatment should be further verified in large-sample, multicenter, prospective studies in the future.

## Data availability statement

The original contributions presented in the study are included in the article/supplementary material. Further inquiries can be directed to the corresponding authors.

## Ethics statement

The studies involving human participants were reviewed and approved by the Ethics Committee of Zhongshan Hospital, affiliated to Fudan University. The patients/participants provided their written informed consent to participate in this study.

## Author contributions

DL, PL, and WZ contributed equally to this work and share first authorship. DL, PL, and WZ conceived and designed the study. DL and PL analyzed the data. DL, PL, and WZ prepared the figures and wrote the main manuscript. JL,HW, and XH provided technical guidance. XH and JG revised the manuscript. DL, PL, and XH provided funding support. All authors reviewed the manuscript and approved the final version for publication.
